# Exploring the impact of xenobiotic drugs on forensic entomology for accurate post-mortem interval estimation

**DOI:** 10.3389/finsc.2024.1411342

**Published:** 2025-01-28

**Authors:** Sapna Jain, Jonathan J. Parrott, Gulnaz T. Javan

**Affiliations:** ^1^ Department of Physical and Forensic Sciences, Alabama State University, Montgomery, AL, United States; ^2^ School of Interdisciplinary Forensics, Arizona State University, Glendale, AZ, United States

**Keywords:** entomotoxicology, PMI estimation, xenobiotic drugs, substance abuse, drug overdose

## Abstract

Forensic entomotoxicology is an emerging field within forensic entomology that investigates the effects of chemicals, drugs, and toxins on insect development and their implications for postmortem interval (PMI) estimation. This systematic overview delves into the influence of drugs such as Morphine, heroin, Opiates, and cocaine on the variables affecting the use of forensically significant insects as evidence tools. Notably, it has been observed that the presence of drugs does not appear to alter the progression of the lifecycle from the first instar to the emergence of flies, indicating that PMI estimations based on fly emergence remain unaffected by drugs. However, larvae treated with drugs frequently show delayed pupation, suggesting the need for further research into the impact of different compounds on various insect species over more extended observation periods. Additionally, conflicting results have been noted regarding how toxins can influence the developmental process in larvae, underscoring the necessity to assess the effect of different classes of compounds on other insect species. The study also recommends exploring factors such as the samples’ collection site and the drugs’ pathological implications to inspire future research. Furthermore, the paper underscores the potential for varying drug effects across insect species, emphasizing the complexity of interpreting drug impacts on PMI estimations. This systematic review was conducted by the Preferred Reporting Items for Systematic Review and Meta-Analyses (PRISMA) guidelines.

## 1 Introduction

The field of entomotoxicology, a relatively new area, has its roots in forensic entomology research dating back to 1980. The first article discussed the use of phenobarbital in drug abuse cases where the remains of a young woman in advanced decay were found. With no fluids or tissues for analysis, toxicological analysis was performed using insect larvae recovered from the body. The larvae contained a significant amount of phenobarbital, indicating a likely cause of death due to a drug overdose (1). Since then the effect of many drugs (2–5), toxic chemicals, including pesticides (6–9), insecticides (10–13), steroids (14), opioids (4, 5, 15), benzodiazepines (3, 16) and their effect on the development of dipteran larvae or succession of insects have been studied.

PMI estimation is an essential aspect of death investigations involving deceased individuals; however, it suffers from various limitations and inaccuracies arising from the many variables, such as environmental conditions (17–19), temperature, humidity, geographical location, availability of insects, microbiome, and toxins or drugs consumed antemortem (20). The U.S. Department of Health and Human Services (HHS) reported the following findings in the survey (2022) conducted in collaboration with the Substance Abuse and Mental Health Services Administration (SAMHSA) (21): “In 2022, 48.7 million people aged 12 or older (or 17.3%) had a substance use disorder (SUD) in the past year, including 29.5 million who had an alcohol use disorder (AUD), 27.2 million who had a drug use disorder (DUD), and 8.0 million people who had both an AUD and a DUD” (21). According to the Centers for Disease Control and Prevention (CDC), more than 106,000 persons in the U.S. died of drug overdose, including illicit drugs and prescription opioids, in the year 2021 (22). The data underscores the urgent need for developing entomotoxicological approaches and techniques. These are essential in situations of poisoning, drug overdose, or suicide when a body is found in a highly decomposed state, and conventional toxicological evidence-like tissues, fluids, or blood samples are unavailable for forensic examination.

**Figure 1:**
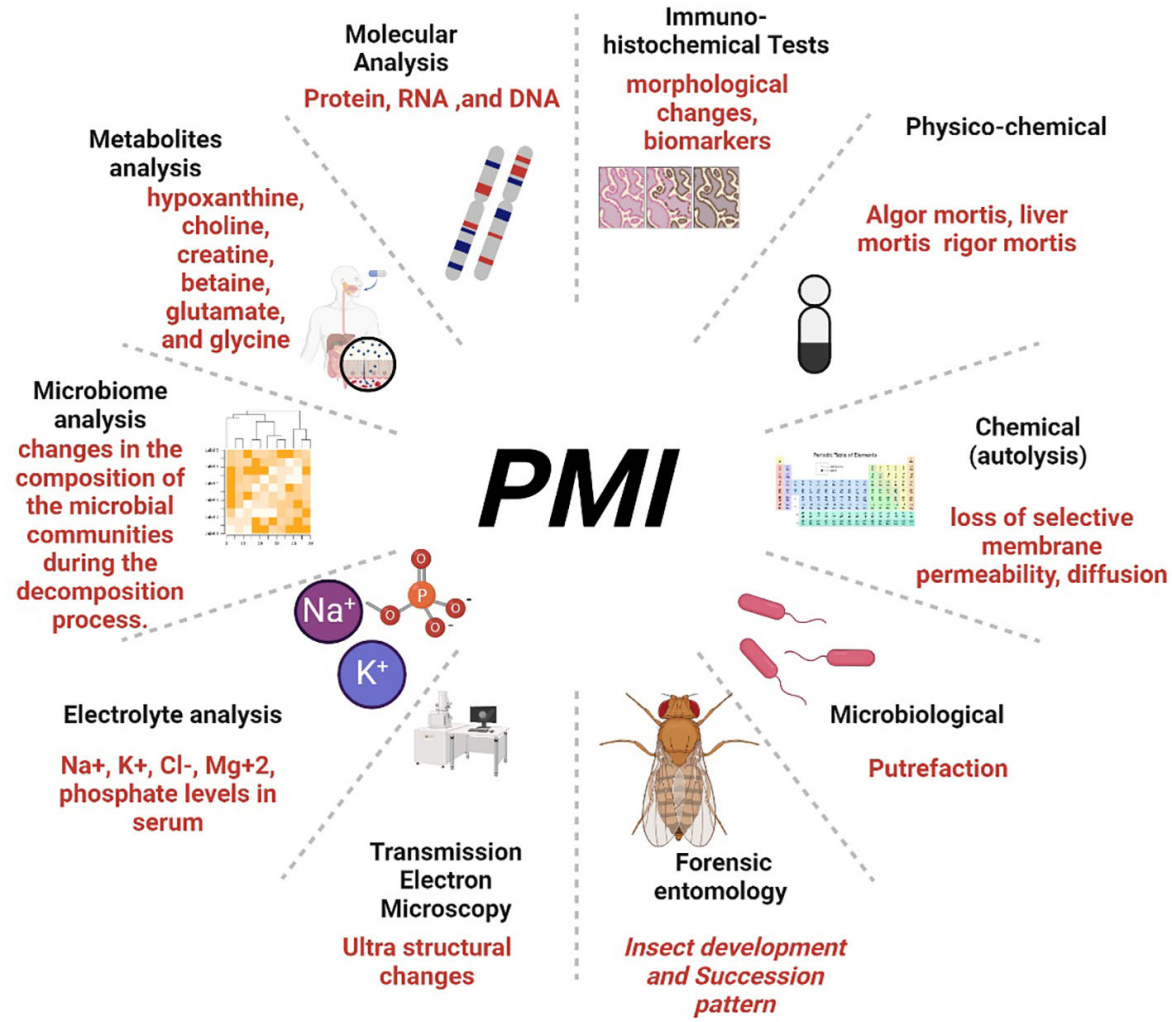
The figure explores a comprehensive overview of PMI estimation methods.

There is no standalone method for accurately determining the exact ‘time of death’ known as Postmortem Interval (PMI) Estimation. Instead, an approximate duration of death is estimated as an ‘interval’ of death, as commonly reported in crime investigations (23). The estimation of the post-mortem interval (PMI) traditionally involves examining changes in the body, including physiological changes (Algor mortis, Rigor mortis, and Livor mortis) (24), metabolic changes (supravital reactions) (25, 26), and the essential role of microbial decomposition (27–30) as illustrated in **Figure 1**. Additionally, forensic entomology, a specialized branch of entomology, utilizes insects as evidence to determine the cause and time of death, employing two approaches based on the developmental pattern and succession of insects during different stages of body decomposition (31–34).

Tomberlin and colleague (35) address issues surrounding the reliability of forensic analysis, specifically biases resulting from media exposure, unconscious biases, and fraudulent data analysis and interpretation. They have outlined a roadmap for improving research and practice to mitigate these concerns. Efforts to undergo next-generation forensic entomology research should include the characterization of phenotypic divergence among fly populations. Studies should be conducted to determine if population identity correlates with variation in phenotype and to characterize the population structure within the species, especially in cases where genetic variation for forensically informative traits is demonstrated. If no correlation between trait variation and population membership exists, attempts should be made to find the genetic variation that correlates with phenotypic divergence. Evidentiary flies should be considered locally informative if populations develop differently and markers for these divergent phenotypes can be found. Prior to estimating their ages using developmental data, flies should be appropriately assigned to their respective populations and/or phenotypic classes. This practice is expected to result in lower error rates for PMI estimates due to a better fit of predicted development rates to true development rates. When genetic variation in forensically informative traits is proven but marker loci cannot be identified, it is important to thoroughly investigate the entire spectrum of expected variation. This includes calculating confidence intervals for predictions based on arthropod evidence (35). Although this approach may result in wider confidence intervals for predictions derived from entomological evidence, they will be more realistic and firmly grounded in basic scientific principles.

According to Byrd et al. (36), there are two approaches to deciding PMI using insects based on the developmental pattern of the insects and based on the succession of the insects during different phases of decomposition of the carcass (31–34). Insects provide an opportunity to determine PMI beyond 72 hours since death accurately. However, several factors may interfere with the accuracy of PMI, such as temperature, pH, and integrity of carrion, which could affect the insect’s life cycle by either decreasing or increasing the time of development and the insect succession pattern (37–42). Another factor that may lead to erroneous determination of PMI estimation is ignoring the effect of drugs on the developmental pattern of insects. The area of forensic entomology that deals explicitly with the impact of drugs and toxins on insects is known as entomotoxicology (43–47).

These findings carry significant ramifications for forensic entomotoxicology and forensic investigations. Alterations in the growth rates of insects due to drug exposure could introduce bias in post-mortem interval (PMI) estimations if the influence of drugs is overlooked. Incorrect PMI estimations might, in turn, mislead investigations and result in justice being compromised. Further research is essential to understand how different compounds affect insect species over extended periods (48). This is crucial for forensic investigators who rely on insect development stages to estimate PMI accurately. Factors such as sample collection site, tissue or organ type, and drug effects must be considered to fully comprehend their impact on insect development. It is important to note that the same kind of drugs may affect different insects differently, especially during the pupal stage. This complexity adds to the challenge of interpreting drug effects on PMI estimations, requiring a careful and nuanced approach to forensic entomotoxicological analysis.

This paper is unique in its systematic overview of the impact of the five scheduled categories of drugs, as described by the U.S. Drug Enforcement Administration (DEA), on the development of larvae or the succession patterns of forensically important insects. The paper also emphasizes the need for careful postmortem interval estimation, considering various variables and explicitly focusing on entomotoxicology’s influence on accuracy. Entomotoxicology remains an under-researched area and holds potential for further exploration of innovative methods and strategies to address the limitations of current approaches. The paper also tackles some of the challenges encountered in this field.

## 2 Methodology

The PRISMA guidelines conducted the current systematic review. This method is a widely accepted and thorough framework for conducting and reporting systematic reviews and meta-analyses in scientific literature research. It offers a structured approach to promote transparency, rigor, and reproducibility in the review process, thus facilitating evidence-based decision-making (49, 50).

In this review, our goal is to investigate the challenges associated with estimating PMI that result from the consumption of drugs or toxins by the deceased prior to death. Substance abuse can impact the behavior and life cycle of forensically significant insects, which are frequently relied upon as evidence in determining the PMI.

Our review included a critical evaluation of studies gathered from databases such as PubMed and ScienceDirect, as well as the Elsevier database, Springer Link journals, government websites, and manual searches through reference lists and search engines like Google Scholar. To ensure comprehensive screening, we used specific keywords to minimize the chance of missing relevant studies. Our study design included original articles, reviews, case reports, comparative studies, and case series while excluding unpublished literature, which resulted in 669 publications. The results were then filtered for publications in English, and on further screening, 43 review articles, 3 books 87 research articles, and 52 publication titles were considered. After the screening phase, 58 publications were assessed as eligible for full-text assessment. Finally,23 articles were added through backward search (analyzing the cited references in the selected articles), resulting in 81 articles in the conceptual review. This search was last updated in August 2024. The following query was used: (Postmortem Interval [Title/Abstract]) And (Forensic Entomology [Title/Abstract]) And (Entomotoxicology[Title/Abstract]) And (Xenobiotic drugs[Title/Abstract]). The information in this search was last updated in August 2024. To conduct the literature review, we extracted the title, authors, journal, year, and type of publication for each paper. We also compared and reviewed the bibliographies of all identified papers to find any additional relevant literature.

EndNote bibliography manager software was used which was immensely helpful in a systematic review using PRISMA reporting criteria. EndNote’s capability to store references and its organizational tools greatly assisted in documenting, reporting, and screening this article. Once the database searches were completed, the records were exported into EndNote. EndNote was extremely helpful in (i) Creating a new EndNote Library, (ii) Exporting references, (iii) Creating groups so the references could be transferred from each database to their respective locations in the article(iv) removing duplicates. 

## 3 Forensic entomotoxicology solves drug-induced death cases

**Figure 2:**
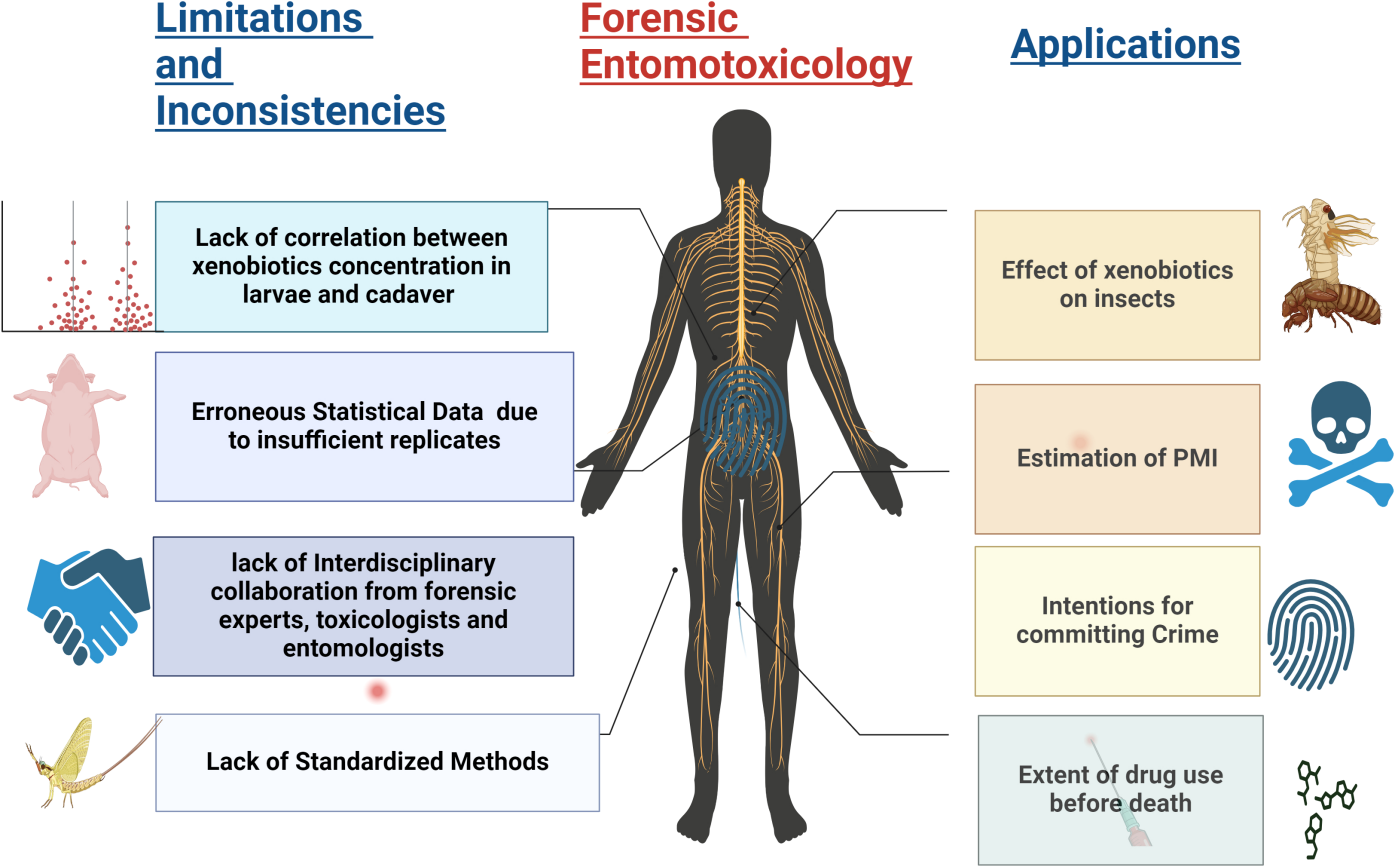
The illustration visually represents the primary application of forensic entomotoxicology. It emphasizes the crucial role played by this field in detecting inconsistencies and underscores the urgent need for a standardized approach.

Forensic entomotoxicology considers the effects of xenobiotics to solve cases of drug-induced deaths when other evidence or postmortem specimens are unavailable. **Figure 2** illustrates the various applications of forensic entomotoxicology studies and how insects can be a surrogate for drug detection when conventional matrices such as blood, urine, or internal organs are unavailable. The figure also highlights the limitations that may influence insect drug detection and provides cues for future research.

In addition, the lack of knowledge of the pharmacokinetics of drugs in insects, the large variability of the experimental set-up, and the toxicological analysis compromise the utility of this science. This section of the paper focuses on the current knowledge of factors influencing insect drug detection and proposes reasons for the limitations and future research recommendations.

Forensic entomologists collect insect larvae from the decomposing bodies to determine PMI. Consumption of any drugs before death may alter the rate of development of the insects feeding on the dead body (43, 51, 52). It may also influence the insects’ succession pattern, *Calliphora stygia* (Malloch, 1781) resulting in erroneous PMI estimations (53–56). This section will focus on how drugs and poisons can affect the growth and development of forensically essential insects and result in inaccurate PMI determination. Entomotoxicology is a subfield of forensic entomology where necrophagous insects are used to determine the presence of drugs in cases of severely disintegrated bodies that do not have soft tissues, organs, or body fluids available for detection of substances that may have caused drug-induced death. In feeding on the cadaver tissues, drugs and toxins present in the tissues also enter the system of larvae. These toxins may further propagate into the food chain through the predators that feed on these larvae, such as rodents or birds. **Table 1** shows how the drugs, chemicals, and toxins affect the developmental pattern of insects, thereby influencing the accuracy of PMI estimation.

**Table 1:** A summary table depicting the impact of drugs and toxins on the larvae of forensically significant insects that infest decaying carcasses.

Family	Species	Drugs/chemicals/ toxins	Drug concentration	Analysis methods	Effect of drugs on larvae	Reference
Calliphoridae	*Calliphora vomitoria* (Linnaeus, 1758)	Cocaine and Heroin	17 mg/kg of cocaine. 34 mg/kg of heroin		Cocaine-fed larvae developed less in length and weight. Heroin-fed larvae showed a more fluctuating pattern, being smaller and lighter.	(100)
Calliphoridae	*Calliphora* *stygia* (Fabricuis, 1781)	Several morphine concentrations were incorporated into pet mince to simulate post-mortem concentrations in morphine, codeine and/or heroin-dosed corpses.	Morphine concentration/g of pet mince: 2 μg/g, 10 μg/g, 20 μg/g	HPLC with acidic potassium permanganate chemiluminescence detection	Growth rates did not differ significantly from those fed on control mince	(110)
Calliphoridae	*Calliphora* *stygia* (Fabricuis, 1781)	Methamphetamine (MA) and its metabolite, p-hydroxymethamphetamine(p-OHMA)	0.1mg/kg MA:1mg p-OHMA; 10 mg/kg MA: 1.5 mg/kg p-OHMA	HPLC–UV	Different temperatures, drug concentrations, and substrate types are also likely to affect the development	(111)
Calliphoridae	*Chrysomya megacephala* (Fabricius, 1794)	Ketamine	50 lg/g ketamine as the LD50 for human being as the oral ketamine LD50 of a 70 kg man is approximately 4.2 g (60 lg/g), and the intravenous LD50 is	GC-MS	ketamine, low temperature, and their synergistic action significantly suppressed the development.	(112)
Calliphoridae	*Chrysomya megacephala* (Fabricius, 1794)	Ketamine	0, 25, 50 and 100 mg/kg	Larvae morphology	The time achieving maximum length and weight was significantly delayed, which results in an increased development duration of larval and prepupal stages,	(113)
Calliphoridae	*Chrysomya megacephala* (Fabricius, 1794)	Malathion	Rabbits were given 0.5, 1.0, and 1.5 times the lethal dose (1.53 mg/kg)of diluted Malathion, respectively, by enema.	Larvae morphology	Duration of the larval and pupal stages was both significantly prolonged	(114)
Calliphoridae	*Chrysomya megacephala* (Fabricius, 1794)	Malathion	The malathion was administered orally with three different doses, which are 10 ml/kg BW, 25 ml/kg BW, and 50 ml/kg BW.	HPLC	Increases in the period of larval development, the maximum length of larvae, and the weight of pupae were observed with increasing malathion concentrations.	(115)
Calliphoridae	*Chrysomya megacephala* (Fabricius, 1794), *Chrysomya saffranea* (Bigot 1877), *Chrysomya rufifacies* (Macquart, 1842) and Chrysomya indiana	Dimethoate	yield 1, 2, 3, and 4 ppm of Dimethoate solutions	Microscopic Morphology analysis	Development of the carrion flies showed a negative correlation with the concentration of the chemical	(116)
Calliphoridae	*Chrysomya megacephala* (Fabricius, 1794), *Chrysomya saffranea* (Bigot 1877)	zolpidem tartrate	1mg/kg-4 mg/kg	Larvae morphology	Weight, width, and length and rate of development of *Ch. megacephala* and *C. saffranea* was negatively affected with zolpidem tartrate concentration	(117)
Sarcophagiadae	*Sarcophaga ruficornis* (Fabricius)	Zolpidem tartrate	1mg/kg-4 mg/kg	Larvae morphology	Zolpidem tartrate retards larval development and alters the estimation of the total developmental duration	(118)
Calliphoridae	*Chrysomya albiceps* (Wiedemann, 1819)	Aluminum phosphide (AIP)(insecticide)	32.8 mg AIP/kg of body weight	Larvae morphology	Larvae body measurements were significantly smaller in the treated group than in the control group	(119)
Calliphoridae	*Chrysomya albiceps* (Wiedemann, 1819)	(AIP)	27.4 mg AlP/kg body weight	HPLC	The AlP concentration in the larvae body was significantly lower than in rabbit tissues.	(120)
Calliphoridae	*Chrysomya megacephala* (Fabricius, 1794), *Chrysomya rufifacies* (Macquart, 1842)	AIP	–	Larvae development	AlP accelerated development until pupation, while the time until emergence remained the same.	(121)
Calliphoridae	*Calliphora vomitoria* (Linnaeus, 1758)	Ceftriaxone and Levofloxacin (Antibiotics)	ceftriaxone and of 2 g/70kg body weight levofloxacin 250 mg/70 kg body weight	Larvae development	Larvae development is delayed by levofloxacin and a mixture of ceftriaxone antibiotics. Both antibiotics had bactericidal effects on the larvae.	(122)
Calliphoridae	*Lucilia sericata* (Meigen, 1826))	Ceftriaxone and Levofloxacin	Tissue concentration of Ceftriaxone 28.57 and Levofloxacin 3.57 μg g^−1^	Larvae development	Delay in the time of pupation high concentration of Levofloxacin. High mortality with both antibiotics.	(123)
Sarcophagiadae	*Sarcophaga peregrina* (Robineau-Desvoidy, 1830)	Ciprofloxacin,	0.111 μg/g -1.33 μg/g	Developmental stages	The growth rate of body length quickened as the concentration of the ciprofloxacin increased. The weight of the pupa and adult was reduced significantly.	(124)

A summary table showing the effect of drugs and toxins on the larvae of forensically important insects that colonize carrion.

Despite the proven dangers of drug consumption, drug usage has gone up by 22% since 2010 (57). It has been projected that by 2030, the number of people using drugs may increase by 11% (57). In the year 2023, the death toll from drug overdose has been reported to be 112,000 according to the Centers for Disease Control and Prevention (CDC) (58). The data indicate that many deaths occur due to substance abuse and sometimes delay in recovering the body may happen due to many reasons such as an individual may have committed suicide at an isolated place and in some scenarios dead body is deliberately concealed. In such cases, it is difficult to determine the cause and time of death by standard toxicological analysis of human specimens therefore, insect larvae can be used as an alternative analyte to detect xenobiotics (45).

Studies have been conducted to determine the effect of drugs on insect development, colonization patterns, and insect succession (37, 45, 46, 54, 59–61). Despite inconsistencies in the methodologies and lack of reliable, replicable data, the information provides useful information for forensic analyses (62, 63).

### 3.1 The influence of drugs on larvae development and insect colonization of the dead body

Insect larval development patterns, particularly in the family Calliphoridae, also known as blowflies, are widely used to determine a minimum PMI estimation because they are usually the first to arrive at a crime scene. Carrion-feeding larvae will also ingest the drugs or toxins in the tissues antemortem, thereby serving as additional or alternate evidence when conventional time-of-death estimation methods cannot be applied. However, reports and studies have indicated that drugs can cause alterations in the development cycle of insects and thereby may interfere with an accurate estimation of PMI (45, 64, 65).

Musvasva et al. (66), reared larvae of *Sarcophaga tibialis* (Macquart,1851) on a metabolite depressant, Sodium methohexital, and a metabolite stimulant, hydrocortisone, at a constant temperature. It was found that the concentration of these drugs did not affect the development time of the larval or pupal stages. There was also no change in the lifecycle of larvae across the test groups from the first instar to eclosion from the puparium (i.e. total developmental time), suggesting that PMI based on the emergence of flies will not be affected. However, larvae treated with either drug took longer to reach the pupation stage.

Wood et al. (67), found that cocaine, heroin, and their combination significantly impacted the developmental times and affected the morphology of *C. vomitoria* larvae. Recently, ketamine hydrochloride and intravenous anesthesia abuse have drawn the attention of the scientific community in China. This study demonstrated that ketamine, low temperature, and their synergistic effects significantly retard the development of the larvae of *Chrysomya megacephala* However, higher concentration did not alter the results significantly. Zou et al. (16), observed the effects of ketamine on the development of *Lucilia sericata* (Meigen,1826). A significant difference in the pathological effect of ketamine was observed in the test group over the control group in terms of the growth of trophocytes in fat body of the larvae. Still, no difference in the length and weight of larvae was noticed among the treated groups. Pathological observation revealed that ketamine could promote the growth of trophocytes in the fat body of *L. sericata* and thereby play a key role on energy storage and utilization and influence biosynthetic and metabolic activity. Another important finding of this study revealed that the effect of ketamine depends on the anatomical site from where the larvae samples are collected. Some of these studies show contradictory results about how toxins could affect the developmental pattern of insect larvae and suggest the need to evaluate the effect of different classes of compounds on different “insects species. Further, a longer observation period is required to assess the impact of other players, such as the site of sample collection from various tissues or organs and the pathological implications of the drugs, which could be an inspiration for future studies. Moreover, different insects could react differently to the same type of drugs, thus concluding the effect of drugs.

The development rate of flesh fly larvae feeding on rabbits treated with heroin (as morphine) was significantly higher than in the control group to alter the PMI estimation up to 29 h (68). Another study conducted by the same group on the larvae of *Parasarcophaga ruficornis* which were grown on tissues from rabbits antemortem fed on methamphetamine, revealed that the development of the larvae was more rapid and expedited the development of larvae by up to 18 h and development of pupae by up to 48 h (69). *Parasarcophaga ruficornis* larvae grown on tissues from rabbits administered with amitriptyline took significantly longer to develop than the control group. The difference in the duration of development of larvae from the control group to the test group was up by 77 h, which is quite significant in altering the PMI estimation (70).

### 3.2 The influence of drug overdose on the succession pattern of the insects

There are two main techniques to determine PMI by using insects: by assessing the development of insect larvae and the succession analysis. The succession pattern analysis is since different species of insects are attracted to the dead bodies at an anticipated time based on the decomposition stage of the carcass. This method of PMI determination is called successional analysis. Successional analysis must be tailored to the specific region as the succession of insects differs by location. Byrd and Tomberlin (71) emphasized that in order to provide a reliable PMI, forensic scientists must compare the insect species discovered from the decomposing body with the database of insect succession for the given region. The type of insect species present on the carcass may provide information about its decomposition stage. However, antemortem drug consumption may influence the successional pattern of the insects and, therefore, may impact the accurate prediction of the PMI. A study conducted in Saudi Arabia on the impacts of antemortem alcohol consumption by domestic rabbits revealed that the successional pattern was not affected. However, there was a delay in the decomposition process by 1-2 days for the carcasses of alcohol-fed rabbits. Ambient temperature impacted the number of insects (4415 insects in winter as compared to 1033 insects in summer). The maximum diversity of the insect population treated with alcohol was 24 taxa. Meanwhile, for the untreated group, 30 insect taxa were observed in winter compared to 26 taxa in summer (72).

Another study revealed similar results that the succession pattern remains unaffected by antemortem consumption of alcohol. This study was conducted on domestic pigs in Blacksburg, VA in the summer of 2023 (73). Results also indicated that there was no apparent difference between the rates of decomposition of the alcohol-treated and untreated pig carcasses despite high concentrations of antemortem blood ethanol.

A study conducted in Iran to study the patterns of succession of insects grown on rabbit carcasses administered with methadone revealed that no significant difference was found between the treated and untreated groups. A slight difference in the pattern of succession of two insect species, *Chrysomya albiceps*, and *Calliphora vicina*, was reported, which could have some bearing on PMI estimation (74).

A study conducted in China from 2015 to 2021 focused on decomposing 18 domestic pig carcasses and the associated insect activity across different months. 53 arthropod species were identified, with varying numbers and species observed in April, June, September, and November. The study found that larvae of family Calliphoridae significantly influenced the decomposition rate, which was highest in June and September, slower in April, and slowest in November. Key species varied by month, with *Calliphora grahami*, *Chrysomya pinguis, Lucilia sericata*, and *Hydrotaea spinigera* being most prevalent in April; *Chrysomya megacephala* and *Chrysomya rufifacies* dominating in June and September; and *Ca. grahami* being predominant in November. Four developmental events of dominant insect species were identified as potential markers to estimate the PMI_min_. The study also observed that insect succession patterns on carcasses varied with the months, offering valuable insights for using insect evidence to estimate postmortem intervals in the Yangtze River Delta region (18).

### 3.3 The effect of drug overdose and its impact on pupae

While it is possible to detect and quantify drugs in adult insects and pupae, conducting these analyses on larvae actively feeding on decomposing material is generally preferable. This preference is due to the requirement for the drug/toxin absorption rate to surpass the elimination rate, allowing for accumulation in the insects. This condition is met when the insects are in the active feeding stage, typically as larvae (72).

In some cases, the stage with the highest accumulation may not always be the larval stage. For instance, in a research study on *Lucilia sericata*, it was found that the larval stage had the highest bioaccumulation factor (BAF) for cadmium (ranging from 0.20 to 0.25). In contrast, the puparial stage accumulated more thallium than the other stages tested (with a BAF ranging from 0.24 to 0.42). This indicates that different substances may lead to distinct bioaccumulation patterns (75).

In an experimental study, *D. Melanogaster* pupae of both genders were divided into experimental and control groups. They were housed in test tubes with bananas and exposed to varying concentrations of morphine. The impact on development stages from fertilization to adulthood was examined, and statistical analysis was performed using SPSS software (76). The results indicate that while morphine positively affects larvae development, it negatively impacts the pupal stage, causing developmental delays and a decrease in the adult fly population due to increased mortality and developmental issues in pupae.

Studying how different substances influence insect development is vital for understanding their implications on PMI estimation. Although it has been observed that the presence of drugs does not seem to alter the lifecycle progression from the first instar to the emergence of flies, indicating that PMI estimations based on fly emergence might remain unaffected, the influence of toxins on the pupal stage cannot be overlooked (77). Conflicting results have been noted regarding how toxins can influence the developmental process in larvae, which directly impacts the duration until pupation.

The findings of a related study explore the impact of the primary metabolites of cocaine and heroin, both individually and when combined, on the developmental pace of C. vomitoria. The results indicate that the metabolites from both cocaine and heroin, as well as their combined form, have a considerable influence on the growth patterns of these insects. Each of the three treatment scenarios changed the insects’ morphology throughout their developmental stages from the first to the third instars, resulting in shorter and lighter specimens. Additionally, these conditions markedly affected the duration of the insects’ life cycles. Treatments involving cocaine and a mix of drugs prolonged the durations of the second and third instar phases but resulted in reduced pupation time and quicker eclosion. On the other hand, exposure to heroin alone extended the pupation period. Notably, the impacts of the drug mixture closely resembled those observed with cocaine alone (67).

### 3.4 Puparia cases as toxicity indicators

Puparial cases are frequently the only remnants found near a deceased body. Even molecular techniques may not be feasible in many cases due to the natural breakdown of DNA, proteins, and enzymes (78).These cases degrade slowly and can be discovered near cadavers, sometimes years after death. These cases are created during the pupation stage from the outer layer of third-instar larvae and can be used as a substitute material for toxicological analysis when live insects and appropriate tissues are unavailable (79).

A study revealed that empty puparial cases of *L. sericata* found on decomposing human remains can serve as an alternative material for detecting Cd and Tl present in the body at the time of death. The accumulation of Cd and Tl in larvae, puparial cases, and adults exposed to different concentrations of these metals in the food substrate was examined. Findings showed that the metal content in larvae, puparial cases, and adults exposed to contaminated liver substrate was significantly higher than that of those exposed to a control substrate, and it increased with increasing metal concentration in the liver. Among the three developmental stages analyzed, the highest average content of Cd was found in larvae, while for Tl, the highest bioaccumulation factor was observed for puparial cases. The accumulation of thallium in these chitinized remnants could be crucial in forensic examinations, as puparial cases can persist near human remains for an extended period (75).

During the larval development stage, determining age is straightforward based on morphological changes, length, and weight variations. However, estimating pupal age is more challenging due to the lack of visible anatomical and morphological changes. Therefore, the development of new techniques and methods is necessary to estimate pupal age through standard experiments accurately (80).

A recent research study revealed that drugs, particularly amitriptyline, were discovered in chitinised insect tissues such as puparial cases and exuviae. These insects were found in association with mummified human remains. Interestingly, the concentration of the drug was observed to be higher in the puparia compared to the beetle exuviae that were analyzed (81).

## 4 Xenobiotic effect of the drugs, substances, and toxins on insects

This section aims to explore the impact of five categories of drugs on insects to address current knowledge gaps regarding whether medications within the same category induce comparable effects on insect development, colonization patterns, and succession. For instance, “morphine, a metabolite of heroin, shares similar pharmacokinetics with other opiates.” This leads to the question: do these similarities extend to their effects on carrion insects? Can we generalize the results based on the drug’s nature, bioactive molecules, and their impact on carrion insects?

Drugs and toxins consumed before death can influence the growth and development of insects that colonize them. Insect development-based PMI estimation, therefore, can result in erroneous results. For example, some drugs, such as cocaine and heroin, in the carcass can accelerate the development of insect larvae feeding on the carcass. Whereas some other drugs, such as malathion and those containing arsenic, can delay it. Heroin, codeine, and methamphetamine speed up the rate of development, thereby interfering with the accurate estimation of PMI based on the length and size of the insect larvae.

An attempt has been made to summarize the results from significant studies of the xenobiotic effects of five distinct categories of drugs, substances, and toxins as identified by the United States Drug Enforcement Administration(DEA) (82). “ The abuse rate is a determinate factor in the scheduling of the drug; for example, Schedule I drugs have a high potential for abuse and the potential to create severe psychological and/or physical dependence.” (82).

Since the first report using entomotoxicological analysis in the medico-legal field by Beyer et al. (83) in 1980, this approach has been widely used in forensic analysis (3, 5, 6, 45–47, 51, 84). Even though entomotoxicology is a widely used and accepted method of forensic analysis, there exist inconsistencies in the results. More research is required to standardize the methodology and effects of the various classes of drugs on insects.

### 4.1 Xenobiotic accumulation of Schedule I drugs in insects

These drugs are purely used for substance abuse and have no recognized medical applications. Some of the drugs in this category include heroin, lysergic acid diethylamide (LSD), marijuana (cannabis), 3, 4-methylenedioxymethamphetamine (ecstasy), methaqualone, magic mushrooms, and peyote (85). It is perplexing that heroin and marijuana are both put in the same Schedule I category belonging to the most dangerous drugs, whereas cocaine is a Schedule 2 drug. So, what are the DEA criteria for scheduling? And does the government think that marijuana is more dangerous than cocaine and meth? One of the main criteria applied in scheduling the drugs is their usage in medical applications and not necessarily the potential danger. Marijuana hasn’t been considered for legal, medical use, and therefore, it is a Schedule 1 drug. However, some studies have suggested the medical applications of marijuana, a derivative of the plant *Cannabis sativa*. The main active molecules present in marijuana are delta-9 tetrahydrocannabinol (THC) and cannabidiol (CBD). THC interferes with brain areas responsible for balance, coordination, memories, and attention. As a result of THC, people get a temporary ‘high’, but on the other hand, there’s a severe impairment of motor and coordination skills. The U.S. federal government has marked marijuana as illegal; however, many states have approved its use as a recreational drug with low concentrations of THC. U.S. Food and Drug Administration (FDA) has approved three drugs with low THC and purified CBD content: Epidiolex, an epilepsy drug, “dronabinol to treat nausea and vomiting from chemotherapy and nabilone to treat low appetite in HIV patients” (86).

The effects of morphine, a heroin metabolite, have been studied on rabbit carcasses and insects feeding on the carcass: *Chrysomya albiceps* (Wiedemann, 1819), which was the most abundant and first to arrive on the body, and *Creophilus maxillosus* (Linnaeus, 1758) (87). Some of the critical observations from this study attested to the utility of entomotoxicology in forensic analysis and the cause of death by analyzing the drugs in insects feeding on the carcasses. Results also support the previous studies that morphine is accumulated in different concentrations in different tissues in the body (88–90) and therefore, it is imperative to consider the insect specimens for analysis that are in the same stages of development. Another significant result indicates that the drug concentration varies at different stages of larval development (70). The concentration of morphine was found to be higher in the feeding stage when larvae feed on the carcass actively and thereby consuming a large amount of the drug along with the carcass tissues, and lower concentrations in the post feeding stage. It is hypothesized that morphine concentration may get reduced with various developmental stages of the larvae because of drug elimination due to metabolic activities (91–93).

It is known that the amount of drug decreases as we go higher up in the food chain. The results from the study were in line with this statement as Morphine was found in higher concentration in *C. albiceps* species as they were the first insects to arrive on the carcass of rabbit. Negligible concentrations of morphine were detected in *C. maxillosus* as they arrive at an advanced stages of decomposition (47, 94, 95), indicating that beetles may not be the best candidates for entomotoxilogical analysis. “Ecstasy” (3,4-methylenedioxymethamphetamine, MDMA) is considered a party drug and is popular among youths as it is used with other substances such as alcohol and marijuana. People feel a sense of euphoria, mental stimulation, and hallucinations. In a breakthrough study involving five cases concerning MDMA and 3,4-methylenedioxyamphetamine (MDA) overdose deaths revealed exciting results regarding postmortem and antemortem concentrations. The study found an increase in the concentration of both MDMA and MDA postmortem regardless of where the samples were collected. It is suggested that there may be a redistribution of these drugs from one organ to another organ, and postmortem concentrations in blood may not be an accurate determinant of the concentrations at the time of death or antemortem. Therefore, these factors need to be considered before estimating the dosage amount that may have led to death (96).

### 4.2 Xenobiotic accumulation of Schedule II opioids in insects

The drugs under this category have a slightly reduced risk of substance use disorder (SUD) as compared to Schedule I drugs. In contrast, they still pose a high potential for misuse and moderate to severe addiction and mental health issues (97). Schedule II opioids include Fentanyl (Duragesic or Sublimaze), Hydrocodone (Lortab, Norco, or Vicodin), Hydromorphone (Dilaudid), Methadone (Dolophine), Morphine (MS Contin), Oxycodone (OxyContin, Percocet, Roxicet, Roxicodone), Opium (97–99). A study was conducted in Iran on a rabbit model(live) where varying dosages of morphine were inoculated in rabbits, and the concentrations of morphine were qualitatively and quantitatively evaluated on the first wave of insects, *Ch. albiceps*, that arrived on the rabbit carcass. Some significant results of the study have indicated that morphine does not accumulate in tissues and is eliminated periodically from the body. There’s no correlation found between the dosage of morphine-inoculated rabbits and the concentration of morphine in insect larvae (87). Heroin and cocaine were found to decelerate the development of *C. vomitoria* larvae, showing a longer time for the second-instar larvae to reach the third instar. This effect may have consequential effects on PMI estimation (100).

Where most scientists have appreciated the usefulness of entomotoxocological analysis in the determination of cause and time of death, Tracqui et al. (101), have a different opinion about the practical applicability of this technique in forensic analysis. Their group studied the effects of toxic compounds such as benzodiazepines, barbiturates, antidepressants, phenothiazine, opiates, cannabinoids, meprobamate, digoxin, and nefopam on 29 carcasses and found no correlation between the drug concentrations in carcass and in insects feeding on them. No reliable results or trends were observed on analyzing the specific organs/tissues of larvae for drugs. The concentration of the drug varied within the anatomical sites of different larvae. Thus, no baseline data could be generated to confirm the reliability of entomotoxicological analysis for cause of death or PMI estimation (101).

### 4.3 Xenobiotic accumulation of Schedule III, IV & V drugs in insects

Schedule III-V drugs include substances or chemicals that have moderate to low potential of abuse and low risk of dependence. These drugs may have medical applications in limited concentrations. A study shows that testosterone (Schedule III drugs) significantly affects the development pattern of *Ch. albiceps* larvae. Even though there was no difference observed in the stages of development, there was a significant increase in weight in the larvae induced with testosterone (102). *Chrysomya albiceps* play a significant role in solving sexual crime cases as they have Y-STR DNA in their gastrointestinal tracts that can provide good evidence for crimes involving rape and death (103).

Both Valium and Xanax (Schedule III drugs) belong to a broader class of drugs, benzodiazepines, commonly prescribed to anxiety patients (104). The prescription drugs that belong to the class benzodiazepines include Valium, Xanax, Halcion, Ativan, and Klonopin (105).

“Valium (Schedule IV drug) is a brand name for diazepam while, Xanax is a brand name of alprazolam. Both drugs act like minor tranquilizers” (104). Alprazolam drug is a psychoactive drug and is used to treat anxiety disorder, social phobias and panic disorders. However it is susceptible to misuse (106). According to a study, alprazolam seem to be relatively benign when taken alone as a therapeutic but may have toxic effects when taken in conjunction with illicit drugs such as ethanol (13%), amphetamine (46%), cannabis (32%), or heroin (14%) (107).

A study investigated the effects of diazepam on the development of *Ch. albiceps* feeding on rabbit carcass (108). It was shown that diazepam accelerates larval growth but did not affect pupal development. The results indicated that the presence of diazepam accelerated the growth and development of larvae, however the pupal stage was not much affected by the presence of drug. The pupal stage was possibly not affected by the drug because of possible accumulation of diazepam in the rigid shell protecting the larvae in the pupa stage, actually not entering the metabolic system of the pupa (109). This theory that pupal cases retain the toxins long after the insects have left the shell and can be used as an alibi to provide entamotoxicology evidence (81). Lorazepam has shown adverse effects on the development of the larvae of the insect *Chrysomya rufifacies.* Different concentrations of lorazepam (1ppm, 2ppm, 3ppm, and 4ppm) were mixed with beef liver and found to have incremental effects on the lifecycle as compared to the control group. The morphology of the insects treated with the drug also decreased as compared to the untreated group. Thus, these results must be considered in determining PMI estimation considering the developmental pattern of the insects.

## 5 Conclusion

This research provides a fresh perspective on forensic entomotoxicology, presenting valuable insights for medical examiners and forensic entomologists. It is imperative to study the effect of various drugs on the developmental pattern of the insects used for forensic analysis and compare it with the baseline data available for accurately calculating PMI. Concentrating on the five drug schedules specified by the DEA emphasizes the significant progress made in this evolving field. The study indicates that while numerous species native to Australasia and Europe have been explored, only a few critical insects relevant to forensics have been scrutinized in the U.S.A. Although essential species have been investigated, further research is warranted to examine additional species of forensic significance, and a broader range of drugs commonly encountered in death investigations. Standardizing this field is critical, and it can be achieved by developing comprehensive standard operating procedures endorsed by peers and the forensic community. This initiative would bring attention to new avenues of research and potential sources of error in the analysis.
